# Implementing the Bloom Trial: Community health nurses’ perceptions of potential barriers

**DOI:** 10.1093/eurpub/ckac131.443

**Published:** 2022-10-25

**Authors:** L Brautsch, L Kierkegaard, RR Carlsson, CT Bonnesen

**Affiliations:** National Institute of Public Health, Copenhagen, Denmark

## Abstract

**Background:**

The overall aim of the Bloom Trial is to develop and test a program promoting healthy weight development during infancy within the community health nurse setting in Denmark. Many interventions are poorly implemented, and to ensure adequate implementation support, previous studies have suggested to assess barriers and organizational readiness before intervention start. The aim of the present study is to assess barriers for implementing and sustaining the Bloom Trial among program adopters and implementers.

**Methods:**

Telephone interviews with managing health nurses (adopters) and health nurses (implementors) from twenty Danish municipalities were carried out (n = 22) in 2017. Moreover, two workshops with health nurses, and continuously meetings with implementation science experts were conducted in 2021-2022.

**Results:**

Barriers were identified on different levels. Organizational barriers within the work of health nurses included lack of time, economic resources, project fatigue, and political priority. Furthermore, health nurses lacked relevant tools to guide parents about promoting healthy weight development. Interpersonal barriers between health nurses and parents were identified as the difficulties of having conversations about healthy weight development, especially if the parents or health nurses were overweight themselves. Cultural differences including language barriers and different perceptions of for example healthy food choices were also found.

**Conclusions:**

The findings are central for ensuring that the Bloom Trial is relevant and applicable to the setting of health nurses in Danish municipalities. This is crucial for ensuring successful adoption, implementation, and prolonged sustainability.

**Key messages:**

• The study pinpoints key barriers (e.g. lack of time, project fatigue and cultural factors) of implementing an intervention promoting healthy weight in the community health nurse setting in Denmark.

• Involving community health nurses’ perceptions aim at increasing the chances of producing a relevant and successful implementation strategy.

